# The Economic Burden of Cancers Attributable to Infection in the Republic of Korea: A Prevalence-Based Study

**DOI:** 10.3390/ijerph17207592

**Published:** 2020-10-19

**Authors:** Thi Xuan Trinh Nguyen, Minji Han, Moran Ki, Young Ae Kim, Jin-Kyoung Oh

**Affiliations:** 1 Department of Cancer Control and Population Health, National Cancer Center Graduate School of Cancer Science and Policy, 323 Ilsan-ro, Ilsandong-gu, Goyang-si 410-769, Korea; xuantrinh93@gmail.com (T.X.T.N.); mjhan@ncc.re.kr (M.H.); moranki@ncc.re.kr (M.K.); 2 National Cancer Control Institute, National Cancer Center, 323 Ilsan-ro, Ilsandong-gu, Goyang-si 410-769, Korea; 12274@ncc.re.kr

**Keywords:** cancer, infection, economic burden, Korea

## Abstract

Infection is a major cause of cancers. We estimated the economic burden of cancers attributable to infection in 2014 in Korea, where cancer causing infection is prevalent, but the economic burden of it has never been examined. Cancer patients were defined as those having made medical claims as recorded by the National Health Insurance Service, which is a mandatory insurance for all citizen. We multiplied the costs by the population-attributable fraction for each type of cancer. The study included direct and indirect costs, where direct costs comprised direct medical and non-medical costs of inpatients and outpatients, while indirect costs were estimated by identifying future income loss due to premature death, productivity loss during hospitalization/outpatient visits, and job loss. In 2014, there were 100,054 infection-related cancer patients, accounting for 10.7% of all Korean cancer cases for that year. Direct costs of cancers associated with infection stood at nearly USD 676.9 million, while indirect costs were much higher at USD 2.57 billion. The average expenditure of a typical patient was USD 32,435. Economic burden of cancers attributable to infection is substantial in Korea, accounting for 0.23% of the national gross domestic product and 1.36% of national healthcare expenditure in 2014.

## 1. Introduction

Cancer places an enormous burden on society regardless of countries’ development. Overall, the numbers of new cancer cases and deaths reported worldwide in 2012 were 14.1 and 8.2 million, respectively [1]. Korean age-standardized cancer incidence rates were among the highest globally at an average of 253.8 per 100,000 population [2], while a remarkable improvement in 5-year survival rates was seen between 1993 and 1995 and in the next 4 years from 2010 to 2014 [3]. In 2015, the national cancer burden was predicted to increase to nearly 280,556 new cancer cases and 76,698 cancer deaths with the increasing population age [4].

A significant number of infectious agents, namely helicobacter pylori (HP), hepatitis B virus (HBV), hepatitis C virus (HCV), human papillomavirus (HPV), *Clonorchis sinensis (C. sinensis)*, Epstein Barr virus (EBV), human immunodeficiency virus (HIV), and Kaposi sarcoma-associated herpes virus (KSHV) have been categorized as Group 1 human carcinogens by the International Agency for Research on Cancer (IARC) [5]. With respect to the global population, the health burden of infection-associated cancers has been recorded on a yearly basis, with 1.9 million cases witnessed in 2002 [6]. Thereafter, the number of cancer cases associated with infectious agents increased to 2 million in 2008 [7] and 2.2 million in 2012 [8], constituting over 15% of all new cancer cases in both years. The proportion of cancer deaths attributed to these infections in low- and middle-income countries was higher relative to that reported for high-income countries; in addition, the proportions of cancer deaths attributed to infection in developed and developing countries were 9% and 20%, respectively [9].

In 2007, the estimated proportions of all cancers in Korea attributable to infection were 25.1% and 16.8% for cancer incidence in men and women, respectively [10]. In addition, those for cancer mortality in men and women were 25.85% and 22.7%, respectively. Furthermore, the proportion of infection-related cancers attributable to infection with HP, HBV, HCV, and HPV was over 97% [10]. Primary and secondary prevention programs to control infections have been implemented. For example, immunization programs against viral hepatitis B and human papillomavirus has been established in 1995 and in 2016, respectively, through the national immunization program, which shows very high vaccination rate [11]. The national screening program provides screening for stomach, liver, colorectal, breast, and cervical cancer for adults in every 2 years, free of charge [12]. Despite those preventable interventions, stomach and liver cancers were predicted to become among the most burdensome for Korean men in 2015, with cervical cancer expected to account for an overwhelming proportion of the cancer burden in Korean women [4]. Importantly, there is sufficient evidence indicating that all these types of cancer are caused by infectious agents.

To date, several cost-of-illness studies have emphasized the large burden incurred by specific types of cancer and groups of cancers linked to risk factors such as smoking [13]. However, no studies have examined the economic burden of cancer associated with infection in Korea. Therefore, research examining the economic cost of infection-related cancer is required to reveal new findings.

## 2. Materials and Methods

### 2.1. Infectious Agents Related to Cancer

The list of infection-causing agents includes HP, HBV, HCV, HPV, *Clonorchis sinensis*, EBV, HIV, and KSHV/HIV. The current study used the population-attributable fraction (PAF), based on previous research conducted in Korea [10].

The total economic cost of cancer due to infection was measured by multiplying the cost of each cancer site by the respective PAF, as described in the following formula:

*Infection-attributable costs = Total costs per cancer site × PAF*

### 2.2. Data Source

Health insurance claims data provided by the National Health Insurance Service (NHIS) were analyzed. NHIS insurance is a mandatory single-payer system that provides benefits for medical services. All South Korean citizens must either enroll in the NHIS (97% of the entire population) or receive medical aid (3%) [14]. The NHIS database contains information regarding both those enrolled in the service and medical aid subjects; therefore, it includes data for the entire Korean population. The NHIS currently maintains and stores national records for healthcare utilization and prescriptions. The NHIS claims data contain details of the cost of care, attended medical institutions, income distribution, and residence for all insurance subscribers [15].

### 2.3. Ethics Approval

This study used anonymous secondary data and was exempted from the Institutional Review Board of the National Cancer Center, Korea (approval date: 13 June 2017; approval number: NCC2017-0131). The requirement for obtaining consent was waived.

### 2.4. Estimation of Economic Burden

We conducted a cost-of-illness study to assess the economic burden of cancer due to infection based on a prevalence-based approach aiming at existing and newly diagnosed patients in 2014. Major cancers attributable to infection were selected with regards to the IARC Monograph Evaluation Carcinogenic Risks to Humans 2012 [16]. Infection-related cancers were defined according to the International Statistical Classification of Diseases and Related Health Problems 10th Revision and included cancers of the oral cavity (C00–C09), oropharynx (C10), nasopharynx (C11), anus (C21), vulva (C51), vagina (C52), uterine cervix (C53), and penis (C60); Kaposi’s sarcoma (C46); Hodgkin’s sarcoma (C81); non-Hodgkin’s lymphoma (C82–85, C96); non-cardia gastric cancer (C161–C168); hepatocellular carcinoma (C220); cholangiocarcinoma (C221 + C240); Burkitt’s lymphoma (C837); and mucosa-associated lymphoid tissue gastric lymphoma (C884) [15]. The target participants were patients who had claimed health insurance benefits with these International Statistical Classification of Diseases and Related Health Problems 10th Revision codes as a primary diagnosis and the special cancer claim code, V193 (a cancer-specific code for expanded benefits), in 2014. Because of the time lag in cancers attributable to infection, the study sample included only patients aged ≥20 years.

The total economic burden of infection-related cancers included direct and indirect costs.

*Total costs = Direct costs + Indirect costs*

Direct costs are costs incurred during medical treatment and consist of direct medical and nonmedical costs. Medical claim records from the 2014 NHIS were used to obtain direct medical care costs, and existing and newly diagnosed patients with infection-related cancer were targeted. The non-covered medical costs were estimated at 19.9% of the total direct medical costs [17]. Direct nonmedical care costs were quantified by acquiring transportation costs for inpatient and outpatient visits and total caregiver payments. One-way transportation fees for inpatient and outpatient visits, obtained from the 2014 Korea Health Panel Survey, were KRW 15,000 and KRW 4000, respectively [18]; these were used with visit frequency, to achieve transportation costs. Based on the 2014 Korea Health Panel Survey, caregivers’ overall daily wage was estimated at KRW 63,000, with a utilization rate of 67% for inpatients [18]. To obtain inpatient caregiver costs, we assumed that each patient was accompanied by a guardian for the entire day; therefore, the cost was calculated by including the daily caregiver payment, duration of hospitalization, and nursing utilization rate. Costs for outpatients aged ≥65 years were determined by multiplying the duration of outpatient visits (in days) by one third of caregivers’ daily wages (see Table A1 in Appendix A).

Formula:

$$\begin{array}{l} \mathrm{DC}\;=\sum_{i}\sum_{j}\sum_{y}\left\{\left(1+\;\propto \right){\;\mathrm{IP}}_{\mathrm{ijy}}+\;\left(1+\propto \right){\;\mathrm{OP}}_{\mathrm{ijy}}\right\}\;\times {\mathrm{PAF}}_{\mathrm{iy}}+\;\sum_{i}\sum_{j}\sum_{y}\left\{\left({\mathrm{IV}}_{\mathrm{ijy}}\;\times \mathrm{TIV}\;\times 2\right)+\;\left({\mathrm{OV}}_{\mathrm{ijy}}\;\times \mathrm{TOV}\;\times 2\right)\right\}\\ \;\times {\;\mathrm{PAF}}_{\mathrm{iy}}+\;\sum_{i}\sum_{j}\sum_{y}\left\{\left({\mathrm{IV}}_{\mathrm{ijy}}\;\times \mathrm{CGR}\;\times C\right)+\;\left(\frac{1}{3}\;\times \;{{\mathrm{OV}}^{\prime }}_{\mathrm{ijy}}\;\times \mathrm{CGR}\right)\right\}\;\times {\;\mathrm{PAF}}_{\mathrm{iy}}\; \end{array}$$

DC = direct costs; j = age; i = gender; y = cancer type

IP = total treatment duration for inpatients of i and j in NHIS data

OP = total treatment duration for outpatients of i and j in NHIS data

α = the ratio of noncoverage and coverage rates (in this study, α = 19.9:80.1)

IV = duration of inpatient visits (in days)

OV = duration of outpatient visits (in days)

TIV = cost of one-way trip to hospital for inpatients

TOV = cost of one-way trip to hospital for outpatients

CGR = caregivers’ average wage per day

C = utilization rate

OV’ = durations of outpatient visits (in days) for those aged ≥65 years

PAF = population-attributable fraction

Indirect costs included future income loss (FIL) because of premature death, productivity loss (PL) due to inpatient or outpatient hospital visits, and job loss (JL) after cancer diagnosis. A human capital approach was applied to the estimate FIL by calculating the income each person would have earned during the period from earlier death to normal life expectancy for the age cohort. A human capital approach based on the assumption that future income acts as a substitute for future productivity was used to determine future income loss, but it does provide an accurate representation in some cases [19]. FIL following premature deaths by year and by cancer types was calculated using data from cause of death statistics [20], and employment rates and annual average wage by sex and age was provided by the Ministry of Employment and Labor [21], with an annual discount rate of 3%. To estimate the loss of productivity due to visits to medical institutions and hospitalization, the total numbers of outpatient visits and days of inpatient admission were obtained from NHIS claims data, which were combined with age and sex-specific employment rates and monthly wages. The rate of job loss due to cancer was estimated at 47%, based on previous research [22]. These figures were used with employment rates and daily average wages according to age and sex, to determine costs incurred due to unemployment following diagnosis (see Table A1). We assumed that the employment rates in age 70–74 years and in age 75 years or older are half of and one quarter of the employment rate in age 65–69 years, respectively, which is the oldest age group available from the Ministry of Employment and Labor.

*Indirect costs = Future income loss + Productivity loss + Job loss*

Formula:

$$\mathrm{FIL}\;=\;\sum_{i}\sum_{j}\sum_{t}\sum_{k=1}^{n}\left\{D_{\mathrm{ijt}}\;\times {\;\mathrm{DPAF}}_{\mathrm{iy}}\;\times \;\left(\frac{{\mathrm{YW}}_{\mathrm{ij}\left(t+k\right)}\;\times {\;E}_{\mathrm{ij}\left(t+k\right)}}{{\left(1+r\right)}^{k}}\right)\right\}$$

FIL = future income loss

i = sex; j = age; t = age at death

y = cancer type

k = 1, 2, …, n (n is the difference between age of death and life expectancy of the age cohort)

D = number of deaths

DPAF = death population-attributable fractions

YW = yearly wage by i and j

E = employment rates by i and j

r = discount rate

$$\mathrm{PL}\;=\;\sum_{i}\sum_{j}\sum_{y}\left\{\left({\mathrm{IV}}_{\mathrm{ijy}}+\;\frac{1}{2}{\;\mathrm{OV}}_{\mathrm{ijy}}\right)\;\times {\;\mathrm{PAF}}_{\mathrm{iy}}\;\times {\;E}_{\mathrm{ij}}\;\times {\;\mathrm{DW}}_{\mathrm{ij}}\right\}$$

PL = productivity loss

i = sex; j = age; y = cancer type

IV = number of inpatient visits

OV = number of outpatient visits

E = employment rate

DW = daily wage

PAF = population-attributable fraction

$$\mathrm{JL}\;=\;\sum_{i}\sum_{j}\sum \left(N_{\mathrm{ijy}}\;\times {\;\mathrm{YW}}_{\mathrm{ij}}\;\times {\;E}_{\mathrm{ij}}\;\times L\;\times {\;\mathrm{PAF}}_{\mathrm{iy}}\right)$$

JL = job loss

N = number of prevalent cancer cases

i = sex; j = age; y = cancer type

YW = yearly wage

E = employment rate

L = job loss rate

PAF = population-attributable fraction

Sensitivity analysis was performed to examine productivity loss following premature death, with 0% and 5% annual discount rates. Furthermore, sensitivity analysis was stratified according to noninsured healthcare costs and the upper and lower bounds of the 95% confidence interval for the PAF. All analyses were performed using SAS version 9.2 (SAS Institute Inc., Cary, NC, USA) and Microsoft Excel 2013. All costs were converted from KRW to USD based on the exchange rate in 2014 (1 USD = 1091.85 KRW) [23].

## 3. Results

There were approximately 100,054 patients with infection-related cancer in Korea in 2014, accounting for almost 10.7% of all cancer cases that year in which no remarkable differences in the figures for males and females were recorded, with relatively equal numbers of patients (approximately 50,000) of each sex (Table 1). Overall, the largest numbers of prevalent cancers attributed to infection were observed in people in their 50s and 60s. With respect to infection types, HP was the most common factor, associated with almost 35% of all infection-related cancers, followed by HBV and HPV, irrespective of sex.

The estimated number of deaths with infection-related cancer was 13,380 deaths, accounting for 13.4% of prevalent cases in that year (Table 2).

The economic burden of cancer attributable to infection in 2014 was USD 3.25 billion, and the majority of the total indirect cost was incurred for HBV, HP, and HCV. The direct cost of cancer attributable to infection in Korean adults aged ≥20 years was estimated at approximately USD 676.9 million (Table 3). The estimated cost incurred during medical treatment was high at almost USD 612.5 million, which accounted for 90.5% of direct costs. Men endured much higher costs, relative to those observed in women, for each infection type, regardless of HPV and HIV status. Noticeably, HBV, HP, and HCV were responsible for the greatest expenditure incurred by men, while HPV accounted for the highest infection-related cancer costs in women, followed closely by HBV and HP.

In contrast, the indirect cost of cancer attributable to infection in Korean adults aged ≥20 years was approximately USD 2.57 billion (Table 3). Regardless of sex, the most burdensome indirect costs were incurred for HBV, HP, and HCV. In particular, the greatest expenses resulting from premature death and lost productivity in men were observed for cancers attributed to HBV, HP, and HCV, while HPV accounted for the highest indirect cost, followed closely by HP and HBV, in women. Economic loss of productivity because of premature death, inpatient/outpatient visits, and unemployment following cancer diagnosis were estimated at USD 1.67 billion, USD 125.7 million, and USD 777.4 million, respectively.

Regarding cancer types, the costs incurred for hepatocellular carcinoma accounted for the largest proportion of the total cost (USD 1.9 billion), followed by uterine cervical cancer (USD 285 million) and cholangiocarcinoma (USD 103 million; Table 4). With respect to infectious agents and sex, the largest economic burden was associated with cancers attributed to HBV in men (approximately USD 1.47 billion), followed by HP- and HCV-related cancers in men and HPV-related cancers in women. The economic burden per patient was highest for cancers related to HBV (USD 48,802) and noticeably greater for Burkitt’s lymphoma and hepatocellular carcinoma, relative to that for other cancer types.

**Figure A1:**
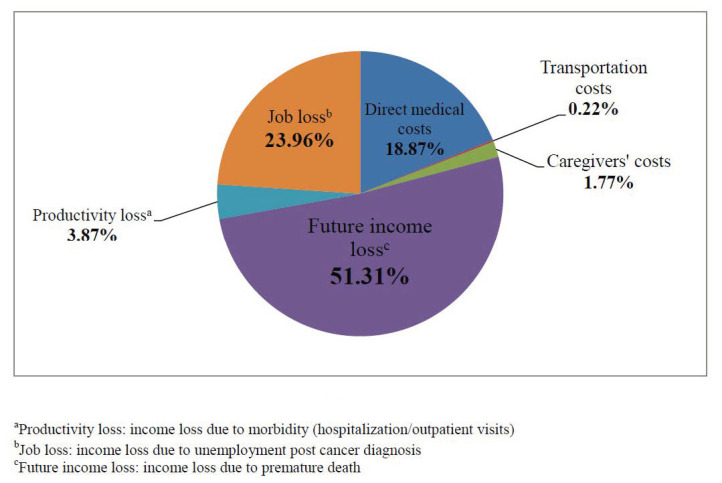
Proportions of cost components.

The largest burden resulting from the economic costs of infection-associated cancers was due to potential future earnings lost because of premature death (51.3%), followed by job loss (24.0%) and direct medical costs (18.9%) (Figure A1).

The results of the sensitivity analysis performed to examine different discount rates showed that the economic burden was USD 3.69 billion with a discount rate of 0% and USD 3.04 billion with a discount rate of 5%. The sensitivity analysis involving the lower and upper bounds of PAF showed that the economic burden ranged from USD 2.68 billion using the lower bound to USD 4.14 billion with the upper bound (Table A2).

## 4. Discussion

The economic burden of cancers attributed to infection in Korea in 2014 was approximately USD 3.25 billion, and indirect costs were 3.8 times higher than direct costs. Indirect costs were significantly more burdensome and 2–3 times higher than direct costs reported in previous studies [13,24]. Furthermore, the financial health burden of cancers attributed to infection accounted for 0.23% of the national gross domestic product and almost 1.36% of national healthcare expenditure in 2014 [14,25].

Regarding direct costs, HBV, HP, and HCV accounted for the majority of infection-related cancers in men, while women’s direct costs were incurred for HPV, HBV, and HP. Trends for indirect costs incurred for the top three infectious agents were similar for both sexes. Overall, HBV accounted for the greatest economic burden of morbidity and mortality resulting from infection-related cancers, followed by HP and HCV. The costs incurred by men for infection-related cancers were four times higher than those incurred by women.

The current study included the cost of job loss as an essential part of indirect costs. Although several Korean studies involving economic burden estimation did not examine income lost through unemployment following cancer diagnosis, job loss should be considered carefully because of the high proportion of the total cost attributable to this factor. The current results suggested that 23.96% of the overall infection-related cancer costs were attributable to job loss. The annual decrease in the mortality rate since 2002, combined with the improvement of 5-year survival rates in cancer patients in Korea [4], raises questions regarding the impact of cancer on patients’ daily lives including employment and work capability. Of the 5396 patients assessed at baseline, 47% lost their jobs over the 3-year follow-up period [22]. This was the primary reason for the inclusion of job loss as a fundamental component of indirect costs in the current study.

From the results of our study, a combination of productivity loss and job loss burden accounts for a substantial fraction of the total infection-related cancer costs (27.8%). There is also other research placing an importance on the burden of productivity loss and job loss in terms of cancer costs. In a study on the economic burden of cancer survivorship among adults in the US [26], indirect morbidity costs were estimated from lost productivity as a result of employment disability, missed work days, and lost household productivity. In particular, mean adjusted annual lost productivity among adults recently diagnosed with cancer was 4694 USD, compared with 17,170 USD of medical expenditure, taking up around 21.5%. In a study on the economic burden of lung cancer and mesothelioma due to occupational and para-occupational asbestos exposure in Canada [27], both human capital and friction cost approaches are employed to determine lost productivity due to cancer, which constitutes 24.4% of total cancer costs. In a Japanese study on the cost of illness of breast cancer trends and future projections [28], productivity loss (morbidity costs) is included, consisting of costs associated with inpatient and outpatient care, holding nearly 6.7% of total costs in 2011 (46.8/697 billion JPY).

In our study, we selected patients based on the special code V193 claimed by medical providers for inpatient stays and outpatient visits. The main reason for this was that, in accordance with the policy of expanding benefit coverage, special codes are provided for patients with severe diseases, such as cancer and myocardial infarction, to reduce the substantial healthcare expenses that could be incurred. In contrast, with the exception of one study that estimated the economic burden of metabolic syndrome-related cancers [29] and used special code V193, studies examining the economic burden of cancer in Korea have defined cancer patients based on the frequency of visits (i.e., at least one inpatient admission and three outpatient visits) [30]. According to the above-mentioned study, there was a slight discrepancy between the economic cost calculated using the frequency of visits and that observed for the special code. Consequently, the use of the special code, V193, in the current analysis could have led to slight underestimation of the total cost; however, this would not have been substantial.

Measurement of the economic burden of cancers associated with infection has not received a considerable attention in developed countries, because these types of cancer are uncommon in highly industrialized nations, with the proportion of cases fluctuating between 5% and 10% [31], while nearly 20% of cancer cases and deaths in Korea have been associated with infection [10]. Many infection-related cancers, particularly those associated with HBV, HCV, HP, HPV, and liver flukes are primarily or secondarily preventable through vaccination, health education for safe sex, changes in eating habits, and early screening. The implementation of the HBV vaccination, for example, started to be packaged into the National Immunization Program in Korea in 1995 [11]. Moreover, there was a marked decline, from 4.6% in 1998 to 2.9% in 2016, in the seroprevalence of hepatitis B surface antigens. Although HPV vaccines have been used in Korea since 2007, HPV vaccination was involved in the National Immunization Program for girls in 2016 [11]. A recent study in Korea reported that, while the prevalence of anogenital warts in men increased continuously over time, that observed in women declined after 2012, reflecting the effect of the vaccine [32]. Korea has adapted its National Cancer Screening Program for the five major types of cancer (i.e., stomach, liver, colorectal, breast, and cervical) since 1999 [12]. However, considering the time lag involved, the long-term effects, including reduced economic burden because of primary and secondary prevention, require sufficient time for examination.

The study was subject to several limitations. The first was the potential overestimation of productivity loss based on the human capital approach used to measure future income loss, as this method has been criticized for its assumption that workers cannot be replaced, even if unemployment is significantly high. However, the human capital approach is one of the most widely used methods in the estimation of indirect costs of diseases. In addition, medical costs could have been underestimated, because the outpatient pharmaceutical costs of cancer-specific treatment could not be verified via the NHIS claim data, and the cost of alternative and complementary medicine was excluded because of an absence of data. Furthermore, cancer costs attributed to infection could have been underestimated, as only primary diagnoses were included in the study. Despite these limitations, this was the first study to focus on the estimation of the economic burden of infection-related cancers in Korea, using NHIS claim data for the entire Korean population. Further investigation on economic burden of infection-specific cancer for evaluating impact of intervention programs of infection control and prevention is needed.

## 5. Conclusions

In the current study, we calculated the economic cost of cancers attributed to infection in Korea in 2014, using a representative dataset from the NHIS, which reflects the entire Korean population. Despite a gradually decreasing trend in the incidence of infection, the results showed a substantial economic burden resulting from infection-related cancers, and this is expected to increase in the near future because of population growth and the time lag between infection and cancer development. These results could assist policymakers to reinforce and promote infection prevention programs and early treatment for infectious diseases nationwide. In addition, the findings could provide a useful baseline for further research examining the effectiveness of ongoing preventive policies in Korea.

## Figures and Tables

**Table 1:** The estimated number of cases of infection-related cancers according to infectious agent in 2014, South Korea.

Infectious Agent	Gender	Age							Total N
		20–29	30–39	40–49	50–59	60–69	70–79	80+	
HP	Men	38	370	2077	6041	7398	6228	1503	23,655
	Women	72	441	1555	2651	2673	3188	1223	11,803
HBV	Men	24	341	2455	8266	7896	5110	1158	25,250
	Women	18	118	442	1596	2299	2340	791	7604
HCV	Men	15	92	575	1881	1783	1147	256	5749
	Women	13	48	147	477	656	659	218	2218
HPV	Men	6	18	47	146	182	170	66	635
	Women	237	2454	4832	5183	3373	2443	857	19,379
*C. sinensis*	Men	0	3	27	125	236	298	109	798
	Women	0	1	6	26	55	91	56	235
EBV	Men	110	150	287	491	368	195	32	1633
	Women	72	85	131	168	98	89	28	671
HIV	Men	4	7	13	23	22	19	5	93
	Women	5	14	26	38	31	28	9	151
KSHV/HIV	Men	3	3	12	12	25	56	32	143
	Women	0	1	1	12	6	8	9	37
Total N		617	4146	12,633	27,136	27,101	22,069	6352	100,054

Abbreviations. HP, helicobacter pylori; HBV, hepatitis B virus; HCV, hepatitis C virus; HPV, human papillomavirus; *C. sinensis*, *Clonorchis sinensis*; EBV, Epstein Barr virus; HIV, human immunodeficiency virus; KSHV, Kaposi’s sarcoma herpes virus.

**Table 2:** The estimated number of deaths with infection-related cancers according to infectious agent in 2014, South Korea.

Infectious Agent	Gender	Age							Total N
		20–29	30–39	40–49	50–59	60–69	70–79	80+	
HP	Men	7	38	155	495	593	748	428	2463
	Women	5	59	139	160	138	383	410	1292
HBV	Men	2	89	518	1439	1295	1238	461	5043
	Women	3	17	55	180	281	510	394	1440
HCV	Men	1	20	116	320	286	272	100	1115
	Women	1	5	16	49	76	139	107	393
HPV	Men	0	1	4	10	10	15	13	52
	Women	6	63	154	205	143	233	212	1016
*C. sinensis*	Men	0	1	8	37	66	100	54	267
	Women	0	0	2	7	16	32	30	87
EBV	Men	1	5	11	39	33	26	12	127
	Women	1	2	5	10	8	14	12	52
HIV	Men	0	0	1	1	2	4	2	10
	Women	0	0	1	2	2	4	3	13
KSHV/HIV	Men	0	0	0	0	2	1	3	6
	Women	0	0	0	0	0	2	2	4
Total N		29	300	1184	2953	2951	3720	2243	13,380

Abbreviations. HP, helicobacter pylori; HBV, hepatitis B virus; HCV, hepatitis C virus; HPV, human papillomavirus; *C. sinensis*, *Clonorchis sinensis*; EBV, Epstein Barr virus; HIV, human immunodeficiency virus; KSHV, Kaposi’s sarcoma herpes virus.

**Table 3:** Estimated direct and indirect costs of infection-related cancers regarding infectious agent in 2014, South Korea (Unit: 1000 USD).

Infectious Agent	Gender	Direct Cost				Indirect Cost				Total
		Medical Costs	Transportation Cost	Caregiver Costs	Subtotal	Future Income Loss	Productivity Loss during Hospital Visits	Job Loss	Subtotal	
HP	Men	89,642	1134	8840	99,616	318,892	23,057	226,787	568,736	668,352
	Women	41,025	556	4564	46,145	66,245	4275	40,122	110,641	156,786
HBV	Men	224,517	2141	18,200	244,858	890,949	56,394	279,422	1,226,765	1,471,623
	Women	59,055	599	5976	65,630	41,623	3886	20,688	66,197	131,827
HCV	Men	51,312	487	4092	55,891	198,980	12,823	64,139	275,941	331,832
	Women	17,465	176	1715	19,357	11,592	1181	6354	19,128	38,485
HPV	Men	4353	60	530	4943	6267	1360	5714	13,341	18,284
	Women	91,145	1430	10,073	102,647	76,219	14,645	100,810	191,675	294,322
*C. sinensis*	Men	9535	97	1081	10,714	21,643	1873	5007	28,522	39,236
	Women	2790	28	366	3184	1564	150	383	2096	5280
EBV	Men	13,853	185	1182	15,221	25,900	4959	21,895	52,755	67,976
	Women	4945	66	475	5487	3582	691	3465	7738	13,225
HIV	Men	1036	9	71	1116	1223	205	1053	2481	3597
	Women	1238	13	108	1360	515	115	664	1294	2654
KSHV/HIV	Men	489	9	71	569	96	113	836	1045	1614
	Women	132	2	21	155	7	11	102	120	275
Subtotal		612,532	6993	57,367	676,892	1,665,298	125,737	777,440	2,568,475	3,245,367

Abbreviations. HP, helicobacter pylori; HBV, hepatitis B virus; HCV, hepatitis C virus; HPV, human papillomavirus; *C. sinensis, Clonorchis sinensis;* EBV, Epstein Barr virus; HIV, human immunodeficiency virus; KSHV, Kaposi’s sarcoma herpes virus.

**Table 4:** Estimated cost of infection-related cancers according to cancer type in 2014, South Korea (Unit: 1000 USD).

Cancer Types	Direct Costs	Indirect Costs	Total
Anus	6456	8934	15,390
Burkitt’s lymphoma	1288	1842	3130
Cholangiocarcinoma	33,687	69,265	102,952
Hepatocellular carcinoma	361,129	1,542,072	1,903,201
Hodgkin’s lymphoma	4128	12,903	17,031
Kaposi’s sarcoma	725	1165	1890
MALT gastric lymphoma	5806	17,393	23,199
Nasopharynx	15,308	45,804	61,112
Noncardia gastric	139,954	661,985	801,939
Non-Hodgkin’s lymphoma	7037	10,575	17,612
Oral cavity	1870	5067	6937
Oropharynx	494	1000	1494
Penis	329	1333	1662
Uterine cervix	97,354	187,590	284,944
Vagina	569	599	1168
Vulva	755	948	1703

Abbreviations. MALT, mucosa-associated lymphoid tissue.

## Abbreviations

HP, helicobacter pylori; HBV, hepatitis B virus; HCV, hepatitis C virus; HPV, human papillomavirus; *C. sinensis, Clonorchis sinensis*; EBV, Epstein Barr virus; HIV, human immunodeficiency virus; KSHV, Kaposi’s sarcoma herpes virus; IARC, International Agency for Research on Cancer; KRW, Korean won; MALT, mucosa-associated lymphoid tissue; NHIS, National Health Insurance Services; PAF, population-attributable fraction; USD, United States dollar.

## Appendix A

**Table A1:** Data sources that are used in direct and indirect costs estimation of infection-related cancers in the Republic of Korea.

Type of Costs		Description	Data Sources
Direct costs	Direct medical costs	Medical care covered by NHIS	NHIS claims data (2014)
		Medical care not covered by NHIS	Survey on the Benefit Coverage Rate of NHIS (2014)
	Direct non-medical costs		
	Transportation costs	One-way cost per visit	Korea Health Panel Survey (2014)
		Frequency of inpatient/outpatient visits	NHIS claims data (2014)
	Caregivers’ costs	Days of inpatient admission	NHIS claims data (2014)
		Frequency of over-65-year-old outpatients	NHIS claims data (2014)
		Caregivers’ daily wage and utilization rate	Korea Health Panel Survey (2014)
Indirect costs	Future income loss	Number of cancer specific deaths	Cause of death statistics, Korea (2014)
		Employment rates	Ministry of Employment and Labor, Korea (2014)
		Average annual wage	Ministry of Employment and Labor, Korea (2014)
		Life expectancy	Life tables, Statistics Korea (2014)
	Productivity loss	Frequency of inpatient/outpatient visits	NHIS claims data (2014)
		Employment rates	Ministry of Employment and Labor, Korea (2014)
		Average daily wage	Ministry of Employment and Labor, Korea (2014)
	Job loss	Number of cancer cases	NHIS claims data (2014)
		Job loss average rate	Park J.H. et al. (2008)
		Employment rates	Ministry of Employment and Labor, Korea (2014)
		Average daily wage	Ministry of Employment and Labor, Korea (2014)

**Table A2:** Sensitivity analysis of infection-related cancer costs regarding discount rates and PAF range (Unit: 1000 USD).

Economic Burden for a Given Discount Rate			Economic Burden within PAF Range		
0%	3%	5%	Lower Bound	Base Estimate	Upper Bound
3,689,134	3,245,367	3,038,634	2,682,638	3,245,367	4,139,011

Abbreviations. PAF, population-attributable fraction.
